# Residency and movement patterns of an apex predatory shark (*Galeocerdo cuvier*) at the Galapagos Marine Reserve

**DOI:** 10.1371/journal.pone.0183669

**Published:** 2017-08-22

**Authors:** David Acuña-Marrero, Adam N. H. Smith, Neil Hammerschlag, Alex Hearn, Marti J. Anderson, Hannah Calich, Matthew D. M. Pawley, Chris Fischer, Pelayo Salinas-de-León

**Affiliations:** 1 Charles Darwin Research Station, Puerto Ayora, Islas Galápagos, Ecuador; 2 Institute of Natural and Mathematical Sciences (INMS), Massey University, Albany Campus, Auckland, New Zealand; 3 Rosenstiel School of Marine and Atmospheric Science, University of Miami, Miami, United States of America; 4 Abess Center for Ecosystem Science & Policy, University of Miami, Miami, United States of America; 5 Universidad San Francisco de Quito, Quito, Ecuador; 6 New Zealand Institute for Advanced Study (NZIAS), Massey University, Auckland, New Zealand; 7 OCEARCH, Park City, United States of America; Shark Research Institute, UNITED STATES

## Abstract

The potential effectiveness of marine protected areas (MPAs) as a conservation tool for large sharks has been questioned due to the limited spatial extent of most MPAs in contrast to the complex life history and high mobility of many sharks. Here we evaluated the movement dynamics of a highly migratory apex predatory shark (tiger shark *Galeocerdo cuvier*) at the Galapagos Marine Reserve (GMR). Using data from satellite tracking passive acoustic telemetry, and stereo baited remote underwater video, we estimated residency, activity spaces, site fidelity, distributional abundances and migration patterns from the GMR and in relation to nesting beaches of green sea turtles (*Chelonia mydas*), a seasonally abundant and predictable prey source for large tiger sharks. Tiger sharks exhibited a high degree of philopatry, with 93% of the total satellite-tracked time across all individuals occurring within the GMR. Large sharks (> 200 cm TL) concentrated their movements in front of the two most important green sea turtle-nesting beaches in the GMR, visiting them on a daily basis during nocturnal hours. In contrast, small sharks (< 200 cm TL) rarely visited turtle-nesting areas and displayed diurnal presence at a third location where only immature sharks were found. Small and some large individuals remained in the three study areas even outside of the turtle-nesting season. Only two sharks were satellite-tracked outside of the GMR, and following long-distance migrations, both individuals returned to turtle-nesting beaches at the subsequent turtle-nesting season. The spatial patterns of residency and site fidelity of tiger sharks suggest that the presence of a predictable source of prey and suitable habitats might reduce the spatial extent of this large shark that is highly migratory in other parts of its range. This highly philopatric behaviour enhances the potential effectiveness of the GMR for their protection.

## Introduction

Effective conservation strategies are urgently required to mitigate and reverse the current global declines exhibited by many populations of large sharks [1–3]. Marine protected areas (MPAs) could play a crucial role in the conservation of shark populations by protecting critical habitats for reproduction and feeding [4,5]. However, given the complex life history, high mobility, and broad spatial ranges of most large sharks, the effectiveness of MPAs for these species remains questionable and in need of critical evaluation, especially given that most MPAs are relatively small and were established to protect highly resident teleosts [6–8].

A scheme called ‘triangle migrations’ was proposed by Chapman et al. [9] to describe the spatial structure of coastal shark populations, based on the movements of sharks between nursery grounds and habitats occupied by adults of different sexes, which tend to display spatial segregation for most of the year [10]. The distances between the habitats used during different life stages, together with the tendency of individuals either to stay for long periods (residency) or repeatedly return (site fidelity) to their home areas (i.e., “philopatry”), can therefore structure populations at identifiable spatial scales [9]. Philopatric behaviour is common in sharks [11,12], potentially reducing the spatial distributions of shark populations and allowing MPAs to be effective at smaller scales than previously supposed [12]. Overall, the benefits of MPAs for sharks will depend on the time individuals spend within their boundaries, which can vary by species, life stage, sex, size, and physiological state as well as the level of protection and enforcement afforded in the protected area [5,8,13].

**Fig 1:**
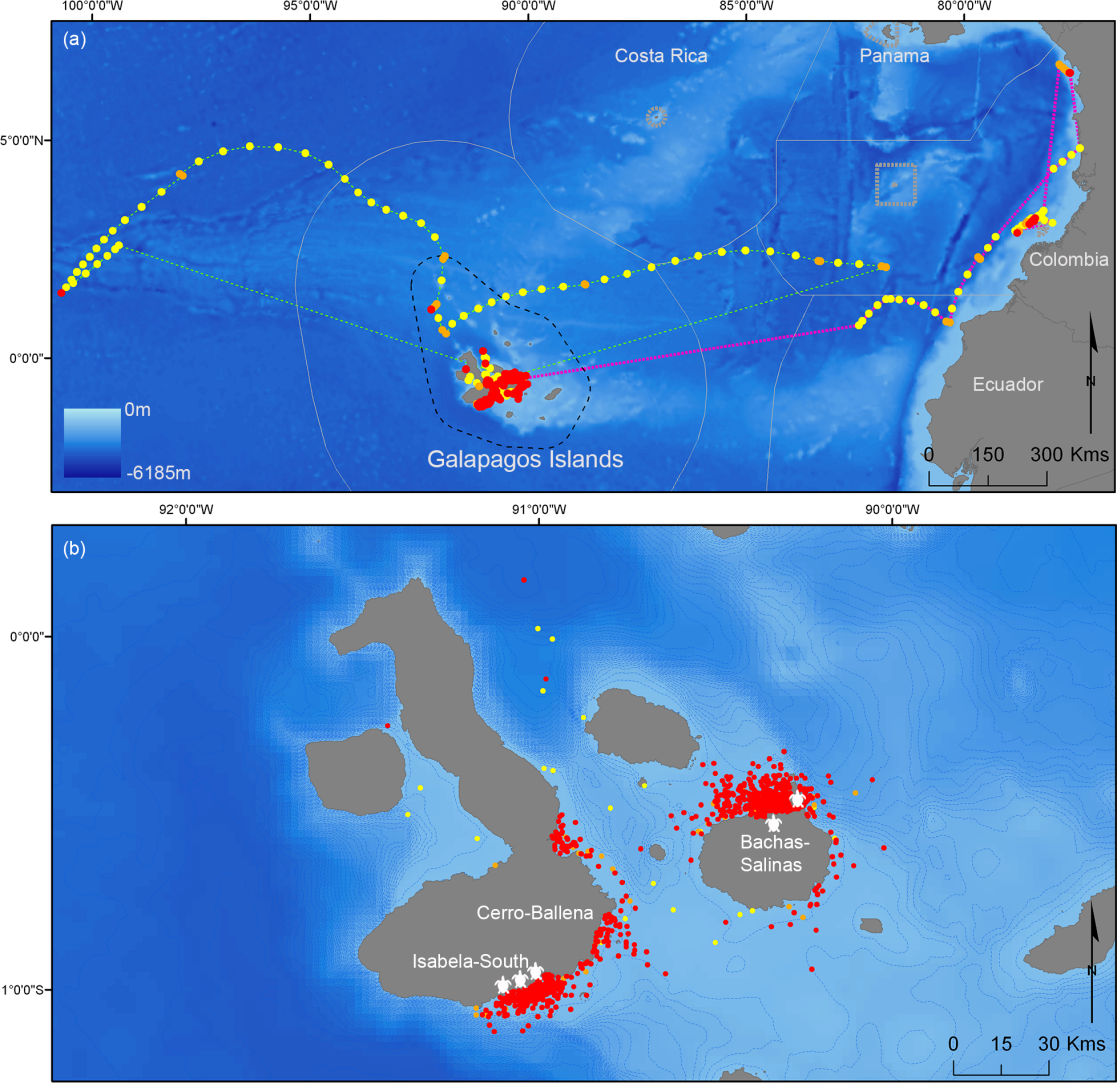
Patterns of residency behaviour of satellite tagged tiger sharks. Resident (red circles), transient (yellow circles) and undetermined (orange circles) behaviours associated with each 12-hour estimated position provided by the switching state-space model. Top panel (a) displays the complete tracks of TS2 and TS4 (pink and green dashed lines, respectively) overlaid with the exclusive economic zones (grey line) and marine protected areas (grey dashed line) of Eastern Tropical Pacific countries. Lower panel (b) shows the estimated positions of all tracked sharks within the Galapagos Marine Reserve (black dashed line, top panel), indicating the study sites, the most important turtle-nesting beaches (sea turtle icons) and the 100 m isobaths (blue dashed lines).

The Galapagos Islands, a Marine Natural World Heritage Site, has been described as one of the richest marine ecosystems in the world [14]. The Galapagos Marine Reserve (GMR; established in 1998) is among the world’s largest MPAs, spanning 138,000 km^2^ [15] (Fig 1A). The GMR harbours abundant populations of marine megafauna, such as large sharks [16–18], with the highest known biomass of sharks in the world in its northern islands of Darwin and Wolf [19]. However, it remains unknown to what extent the spatial ranges of different shark species occur within the GMR.

The tiger shark *Galeocerdo cuvier* (Péron and Lesueur 1822) is a large (up to 381–550 cm total length, TL; see Whitney and Crow [20]) apex predator, globally distributed in coastal and epipelagic waters of temperate and tropical seas [21]. Despite tiger sharks having been observed at the Galapagos Islands since 1924 [22], formal records of tiger shark have been rare at the GMR [16,18]. Tiger sharks display both wide-ranging and resident behaviours, the latter occurring in specific areas with abundant sources of prey [23–25]. It has been suggested that individual tiger sharks learn from experience about the location and timing of such foraging opportunities and may have a mental map allowing them to time their migrations to take advantage of seasonal food pulses [26]. Ontogenetic dietary shifts occur in tiger shark, as young individuals are nocturnal bottom feeders while larger sharks feed on larger prey such as mammals, elasmobranchs and sea turtles [27]. Sea turtles, in particular, have been identified as the most common prey in their diet in some areas of its distribution [28], and large tiger sharks may concentrate their movements around turtle-nesting beaches to take advantage of this seasonally predictable and abundant food source [29–31].

The GMR is one of the most important nesting and resident sites for the green sea turtle (*Chelonia mydas*) in the Eastern Pacific, supporting more than 40% of their total population in this region [32,33]. Close to 2,000 nesting events are recorded annually at the GMR, mostly during the warm season (Dec-May), with peak nesting activities occurring during February and March [33–35].

The present study investigated the spatial ecology of tiger sharks at the GMR, with a particular focus on residency patterns in relation to green sea turtle nesting beaches given their potential importance as an abundant prey item. We employed a combination of field methods, including satellite and acoustic telemetry to record spatial and temporal use of the GMR by tiger sharks as well as stereo baited remote underwater video systems (stereo-BRUVs) to explore differences in the size and relative abundance of tiger sharks at green turtle nesting sites. Specific study objectives were to: 1) describe movement patterns in and around the GMR, 2) quantify degree of philopatric behaviour, including patterns of residency and site fidelity at the study sites, 3) explore ontogenic differences in habitat usage, and 4) test for seasonal differences in relative abundances and size distributions of tiger sharks at the study sites. Based on previous studies [29–31], we hypothesized that tiger sharks would exhibit a high degree of residency and site fidelity to the turtle-nesting areas, and that patterns in the spatial distributions of tiger sharks would also display seasonal, gender-specific and ontogenic variation (following Meyer et al. [26] and Fitzpatrick et al. [29]). We then discuss the implications of our results for the effectiveness of the protection provided by the Galapagos Marine Reserve World Heritage Site to the tiger sharks.

## Materials and methods

### Ethics statement

This research was approved by the Galapagos National Park Directorate (GNPD) as part of the research permit granted to Dr Alex Hearn (GNPD permit #PC-01-14) and Dr. Pelayo Salinas-de-León of the Charles Darwin Foundation (GNPD permits #PC-40-14 & #PC-17-15), with the methods described here reviewed and approved by a Galapagos National Park Directorate’s committee that assesses animal care in research activities.

### Study site

The Galapagos Archipelago is composed of 13 major islands and over 100 islets located on the equator, approximately 1,000 km west of continental Ecuador, in the Eastern Tropical Pacific (ETP; Fig 1A) [36]. Two distinctive seasons occur at the archipelago, driven by oscillations in the strength of predominant currents: a warm rainy season runs from December to May, driven by the northeastern Panama Current; and a cool dry season runs from June to November, due to the Humboldt Current, arising from the southeast [37]. Green turtles nest during the warm season. We gathered data within each of the two distinct climatic seasons, each identifiable by referent to the green turtle-nesting activities: namely, a ‘nesting season’ from December to May (warm period) and a ‘non-nesting season’ from June to November (cool period).

We conducted our study within 2014 and 2015, with sampling focused on three locations in the GMR (Isabela-South, Bachas-Salinas and Cerro-Ballena; Fig 1B). Two locations, Isabela-South and Bachas-Salinas, were selected because they are the two largest nesting areas for green turtles within the GMR [34]. Isabela-South contains several consecutive nesting beaches west of Puerto Villamil, the most important being Quinta Playa, while Bachas-Salinas, located between Santa Cruz and Baltra islands, includes the nesting beaches of Las Bachas and Las Salinas [34,38] (S1 Fig). Both Isabela-South and Bachas-Salinas have similar seabed composition and depth profiles, with predominantly sandy bottoms, sparse rocky reefs, and gentle slopes. However, Isabela-South is exposed to the predominant southern wind and swell, while Bachas-Salinas has more sheltered conditions. The third location, Cerro Ballena, was selected based on the reported incidental catches of juvenile tiger sharks during scientific fishing surveys (Pazmiño pers. comm.). Cerro Ballena is located at the southeastern tip of Isabela Island (S1 Fig), though it is more sheltered than Isabela-South and has no sandy beaches. We focused our sampling efforts throughout the year, encompassing both the green turtle ‘nesting season’ from December to May (warm period) and the ‘non-nesting season’ from June to November (cool period).

### Movement patterns and habitat usage

#### Capture and tagging

Tiger sharks were captured at each of the three study locations (Fig 1B). Sharks were attracted to boats using fish burley and captured using handlines baited with wahoo (*Acanthocybium solandri*) or yellow-fin tuna (*Thunnus albacares*). Following Heithaus et al. [39], captured sharks were secured alongside the vessel and inverted to induce tonic immobility [40], except sharks TS1-4 (Table 1), which were drawn onto a submerged platform attached to a mother vessel (MV Ocearch) that was then raised above the water level. Each shark was sexed and measured, then classified into one of three size classes: small (< 200 cm TL), medium (200–300 cm TL) or large (> 300 cm TL) following Lowe et al. [27].

**Table 1:** Summary of acoustic and satellite tag deployments on tiger sharks at the three tagging locations within the Galapagos Marine Reserve in 2014–15.

	Shark ID	Tagging date	TL (cm)	Sex	Satellite		Acoustic			
					Days transmitting	% residency time*	Days monitored**	Days transmitting	No. detections	RI*** (per tagging site)
Bachas-Salinas										
	TS1	30-Jan-2014	274	F	116	90.48	307	152	761	0.50
	TS2	30-Jan-2014	251	F	210	74.83	79	19	150	0.24
	TS3	30-Jan-2014	248	F	127	99.02	-	-	-	-
	TS4	30-Jan-2014	383	F	333	82.78	262	111	438	0.42
	TS5	11-Jun-2015	225	F	-	-	104	71	482	0.68
	TS6	11-Jun-2015	240	F	67	98.48	104	47	185	0.45
	*Average TL ± SE = 270*.*17 ± 23*.*49*									
Cerro Ballena										
	TS7	23-Jul-2014	140	F	-	-	180	41	140	0.23
	TS8	23-Jul-2014	224	M	25	87.50	303	68	510	0.22
	TS9	24-Jul-2014	234	F	26	100.00	271	16	82	0.06
	TS10	24-Jul-2014	171	F	21	100.00	286	45	399	0.16
	TS11	24-Jul-2014	260	F	115	100.00	-	-	-	-
	TS12	7-Oct-2014	180	M	-	-	113	23	376	0.20
	TS13	7-Oct-2014	180	M	-	-	195	93	1183	0.48
	TS14	21-Feb-2015	206	F	58	78.12	74	10	23	0.14
	TS15	21-Feb-2015	202	M	84	98.30	177	16	99	0.09
	*Average TL ± SE = 199*.*67 ± 12*.*14*									
Isabela-South										
	TS16	22-Feb-2015	378	F	128	100.00	-	-	-	-
	TS17	22-Feb-2015	282	F	14	100.00	58	1	1	-
	TS18	22-Feb-2015	324	M	45	100.00	118	2	2	-
	TS19	23-Feb-2015	286	M	66	95.61	-	-	-	-
	TS20	23-Feb-2015	242	M	37	100.00	68	1	1	-
	*Average TL ± SE = 302*.*40 ± 22*.*93*									

*% residency time refers to the percent of time spent in resident behaviour within the GMR, as determined by the SSSM model.

**Days monitored refers to the number of days that the shark could be detected by the acoustic receivers (note that acoustic receivers were deployed after the sharks were tagged).

***RI = residency index per site (i.e., total number of days a shark was detected divided by the number of days that the shark was monitored by the receivers).

Two types of tagging device were deployed on most of the captured sharks: a satellite SPOT tag was attached to the first dorsal fin (Smart Position or Temperature Transmitting; SPOT5, Wildlife Computers Ltd., Washington, USA; [41]), and an acoustic transmitter was surgically implanted into the intraperitoneal cavity (V16-6x, VEMCO Ltd., Nova Scotia, Canada; see Meyer et al. [23]). Using two tagging approaches provided distinct and complementary information on the movements of tiger sharks at different spatial scales [25].

#### Residency, site fidelity and broad movements from the GMR

The SPOT satellite tags provided geolocations of sharks derived from Doppler-shift calculations made by the Argos Data Collection and Location Service (www.argos-system.org; for a detailed description of the functioning of satellite tags see Hammerschlag et al. [41]). Given the irregularity of positions from SPOT-derived data, spatial analyses were conducted using a Bayesian state-space model (SSM) [42] implemented with the R package ‘bsam’ [43]. As outlined in Jonsen et al. [44], SSMs are “time-series models that allow unobservable, true states to be inferred from observed data by accounting for errors arising from imprecise observations and from stochasticity in the process being studied”. Specifically, SSMs combine a statistical observation model that deals with Argos satellite telemetry precision, with a specified process model of the movement dynamics related to the animal behaviour and environment [45]. This modelling approach offers multiple advantages, particularly when working with diving animals that surface briefly and irregularly; such animals generally yield poor-quality, intermittent tracking data [46]. Analysis by SSMs provides regular estimated positions assuming a correlated random walk on the differences in subsequent locations, rather than on the locations themselves [46]. SSMs also account for the mean turning angle and autocorrelation in speed and direction of the animal, as well as the location error due to the quality of the transmission (modelling the Argos position errors–6 quality classes–with appropriate independent *t*-distributions) [42,46].

To ensure our analyses were as robust as possible, we took several data-preparation steps prior to fitting the model. The data were checked for obvious errors, removing duplicate data points from the same track with the same time and/or position. In addition, tracks with gaps exceeding one week were split into separate segments and recombined after fitting the model (as recommended by Jonsen pers. com.).

We used a hierarchical, first-difference, correlated, random-walk, switching SSM (hDCRWS) [43]. This model allows for estimate parameters jointly across multiple individual tracks. The model provided a set of estimated positions for each shark at regular 12-h time intervals. An interval of 12 h was chosen (following Lea et al. [47]) because the majority (82.2%) of gaps between input points were less than 12 h apart (S2 Fig). Points estimated to be on land were discarded, as were those points that were estimated over intervals lacking data for longer than 3 days (Jonsen pers. com.). The SSM allows for individuals to switch between two behavioural states: an area-restricted search or “resident” state, and a migratory or “transient” state. The behavioural state is inferred based on the simplifying assumption that animals travel in a straight line between regularly spaced unobserved locations and that the spatial autocorrelation among pairs of points is higher when the animal is in a resident state, while turning angles should be closer to 0 in transient states [46]. The behavioural state (*b*_*t*_) is either 1 (resident) or 2 (transient) for each animal at each time point (*t*). Uncertainty in the value of *b*_*t*_ was quantified with a Markov Chain Monte Carlo (MCMC) algorithm. The mean value of *b*_*t*_ across MCMC draws for each animal at each time point was used to classify the state as being either predominantly transient (mean *b*_*t*_<1.25) or predominantly resident (mean *b*_*t*_>1.75) [29,46], with the remaining (5.75%) middle values omitted for the spatial analyses. The relative frequencies of the two behavioural states were then used to evaluate the prevalence of resident *vs* transient states in tiger sharks inside and outside the GMR.

#### Core ranges and activity space

We identified high-use areas for tiger sharks by applying spatial kernel density estimation (KDE) [8,24] to the estimated positions provided by the SSM, pooled across individuals. KDE was conducted in ArcGIS 10.3.3. (ESRI 2016), and was based on the quadratic kernel function described by Silverman [48]. We calculated percent-volume contours (PVCs) using the “isoline” tool available from the Geospatial Modelling Environment add-on to ArcGIS [49]. We defined “core range” (CR) and "activity space” (AS) as the area within the 50% and 95% PVCs, respectively and independently of a track’s duration. Following Hammerschlag et al. [30] we also reported intermediate PVCs (75%). The individual CR and AS data were right-skewed in their distributions so further analyses were based on log-transformed values. Log-transformed CRs and ASs each showed a marginally significant positive linear relationship with the log-transformed number of positions (log-N, where N is the number of positions) from which they were calculated (regression on log-N; coefficient for log-HR: $\hat{\beta }$ = 0.924 ± 0.41 SE, *t*_14_ = 2.24, *p* = 0.042; and for log-CR: $\hat{\beta }$ = 0.744 ± 0.350, *t*_14_ = 2.13, *p* = 0.052). Hence, log-N was included as a predictor in subsequent linear models to account for the length of time over which the individual was observed. Multi-way ANOVA was used to test for variation in AS and CR based on the factors of season and size, with differences in variances evaluated using Levene’s Tests.

#### Habitat usage at study sites

To test for potential ontogenic differences in the associations between tiger shark positions and sea turtle-nesting sites, we conducted chi-square tests on the proportions of the estimated shark positions provided by the SSM of different size ranges that fell within 5 and 10 km of the sea turtle-nesting beaches. In previous studies, a proportion of the sea turtles remained resident within a 10 km range of the beach after nesting [32,35]. The smaller buffer of 5 km was included to explore hierarchical spatial uses of the nesting areas by the different size classes of sharks.

Four acoustic receivers (VR2W, Vemco Ltd., Nova Scotia, Canada) were deployed at the green turtle-nesting beaches (S1 Fig) to measure patterns of residency and site fidelity from acoustically tagged sharks at high spatiotemporal resolution (for a detailed description of passive acoustic tracking see Meyer et al. [23]). One receiver was deployed at each of Isabela-South and Cerro-Ballena from October 2014 to August 2015. One receiver was deployed at each of Las Salinas and Las Bachas from November 2014 to September 2015. Due to the close proximity of Las Salinas and Las Bachas, the data from these two receivers were pooled and analysed as one location (Bachas-Salinas).

Data obtained from the four acoustic receivers were pre-processed, removing single detections potentially caused by signal collisions or noise (following Bond et al. [50]). A Residency Index (RI) was defined, for each shark, as the proportion of the total number of monitored days that the shark was detected (and thus near the nesting beach). This proportion was calculated separately for each season and location (Cerro-Ballena and Bachas-Salinas). Diel patterns of usage were examined by classifying the detections at each location into hourly bins and analysing the counts per bin using a Fast Fourier Transformation (FFT, periodogram function, R package TSA [51–53]. While we did not conduct range testing of receivers, we assumed them to be up to 300 m, based on previous detection ranges obtained in telemetry studies at the GMR [54].

### Size structure and relative abundance at study sites

Data on the presence, relative abundance, size- and sex-distribution of tiger sharks were collected using stereo-BRUVs between March and September 2015. The systems consisted of two GoPro HERO4 digital cameras (GoPro, Inc., California, USA) mounted 0.7 m apart and converging inwards at a 6° angle on stainless steel frames, baited with *ca*. 800 g of yellow-fin tuna (*Thunnus albacares*). Replicate stereo-BRUV deployments were conducted at each of the three locations (S1 Fig) at *ca*. 25 m and at each of two depths: ‘benthic’ sets were deployed 1.5 m above the sea bed [55], and ‘pelagic’ sets were deployed in mid-water at *ca*. 10 m depth [56]. Four benthic deployments were made at Cerro-Ballena, four benthic and four pelagic deployments at Isabela-South, and eight benthic and eight pelagic deployments at Bachas-Salinas during each season (S1 Fig). BRUVs were deployed along the *ca*. 25 m depth contour separated by a minimum distance of 500 m (following Santana-Garcon et al. [56]), alternating between benthic and pelagic deployments. All deployments were made during daylight hours and never within 1 hour of sunrise or sunset. Stereo-BRUVs were set for at least 100 min, with initial and final 5 min periods discarded to minimise the influence of the boat. The remaining 90 min of footage were analysed using the software EventMeasure (SeaGIS Pty Ltd., Victoria, Australia). To evaluate relative abundance of tiger sharks from stereo-BRUVS, we employed an approach modified from Cappo et al. [57]. First, we recorded the maximum number of individual tiger sharks observed in a single still video frame throughout the 90-minute deployment (i.e., MaxN [57]). Next, we added to this value any other tiger shark clearly distinguishable within the deployment that was not already included in the MaxN calculation (i.e. MaxN *plus* number of different tiger sharks identified in deployment). We termed this value corrected MaxN (cMaxN). Different tiger sharks could be distinguished in BRUVS using a combination of several criteria: (1) the presense/absence of claspers in adult individuals, (2) presense/absence and location of scars or markings; and (3) body total length (taken as an average of three measurements of TL, each from a different video frame). Multi-way ANOVA was used to test for variation in lengths based on the factors of sex, season, and location. Seasonal or gender differences in spatial patterns of occurrence were tested using Fisher’s exact tests.

## Results

### Movement patterns and habitat usage

Of the 20 sharks captured (13 females, 7 males; Table 1), 16 were double tagged with both satellite and acoustic transmitters, two were tagged with only a satellite transmitter, and two were tagged with only an acoustic transmitter. All tagged sharks provided at least one type of data (either acoustic or satellite) for a minimum of 14 days after being released, so there was no indication of mortality caused by the tagging process. Two satellite tags (TS12 and TS13) provided no signal, but the acoustic tags of these two sharks provided data for 113 and 195 days, respectively. Two sharks (TS3 and TS19) were never recorded by any of the acoustic receivers. The satellite track of TS19 did not pass close to any of our receivers, but TS3 provided satellite positions for more than 100 days around her tagging site (where the acoustic receivers were deployed), pointing to a likely failure of TS3’s acoustic transmitter.

#### Residency, site fidelity and broad movements from the GMR

When pooled across the 16 sharks for which we received data from satellite tags between January 2014 and July 2015, 1,339 (92.6%) of the 1,446 SSM-estimated positions were located within the GMR. The behavioural state was classified for a total of 1,347 positions, the majority of which (80.6%) were classified as resident. For positions located within the GMR, resident behaviour was also dominant (86.4%). In contrast, 71.0% of positions estimated to be outside the GMR were classified as having transient behaviour, with only 8.4% showing resident behaviour (based on two sharks, TS2 and TS4; see Table 1).

Two satellite-tagged sharks left the GMR during the study period, both of which were female. The first (TS2; 251 cm TL) headed east towards the continental coast of South America in April 2014, exhibiting a mixture of resident and transient behavioural states along the coast of continental Ecuador and Colombia during the following 3 months (Fig 1A). This shark was then detected again at her tagging site by the acoustic receivers at Bachas-Salinas at the beginning of the subsequent turtle-nesting season (S3 Fig). The second shark to leave the GMR was the largest tagged in this study (TS4; 383 cm TL); after transmitting from within the GMR from January–July 2014, her signal was lost, resuming 2 months later in the Pacific Ocean *ca*. 1,000 km west of her original tagging site (Fig 1A). TS4 then headed eastward, passing through the GMR and continuing on to continental South America, when transmissions again stopped in mid-October 2014. One month later, at the beginning of the subsequent turtle-nesting season, TS4 returned to her original tagging site in the GMR at the turtle-nesting beach of Bachas-Salinas (Fig 1A and S3 Fig).

#### Core ranges and activity space

Core range (CR) areas for individual sharks ranged from 2.3–292.5 km^2^, with a median of 73.8 km^2^. Activity space (AS) areas ranged from 11.1–4,976 km^2^, with a median of 174 km^2^.

**Fig 2:**
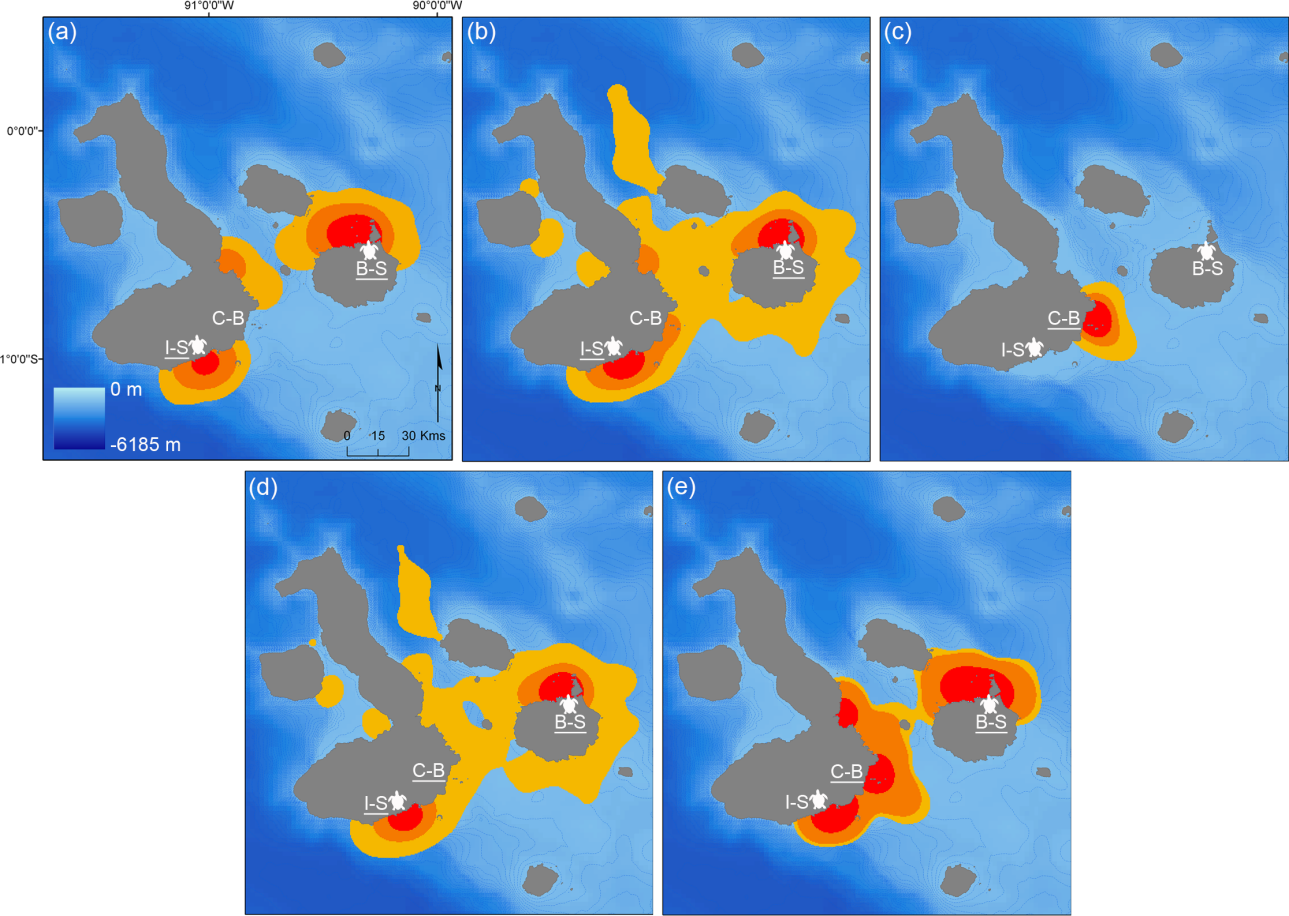
Kernel density estimates of satellite-tagged tiger shark positions. Sharks are pooled by size classes: (a) large (> 300 cm TL, n = 3), (b) medium (200–300 cm TL, n = 12) and (c) small (< 200 cm TL, n = 1); or by season: (d) sea turtle-nesting season (n = 11) and (e) non-nesting season (n = 7). Red indicates core range areas (50% percent-volume contour, PVC), yellow represents activity space areas (95% PVC) and orange indicates the intermediate 75% PVC. Underlined names of study sites (Isabela-South = I-S, Cerro-Ballena = C-B, Bachas-Salinas = B-S) indicate those locations where sharks were tagged in each case. White sea turtle icons show the turtle-nesting areas and local bathymetry is displayed by 100 m isobaths (blue dashed lines).

At the individual level, neither log-AS nor log-CR was significantly related to either season (log-AS: *F*_1, 11_ = 0.67, *p* = 0.431; log-CR: *F*_1, 11_ = 0.03, *p* = 0.872) or TL (log-AS: *F*_1, 11_ = 0.43, *p* = 0.523; log-CR: *F*_1, 11_ = 0.01, *p* = 0.942). There was significantly greater variability in log-AS values during the nesting season (median: 455 km^2^; min–max: 11–4,976 km^2^) *vs* the non-nesting season (median: 118 km^2^; min–max: 81–168 km^2^; Levene’s Test, *F*_1, 14_ = 4.78, *p* = 0.05). There was no such seasonal difference in the variance of log-CR (Levene’s Test, *F*_1, 14_ = 0.23, *p* = 0.64). When points were pooled across individuals within size classes, the collective AS area of medium sharks was approximately double that of large sharks, while the collective CR areas for these two size classes were almost the same (Table 2; Fig 2). When pooled within seasons, different patterns were apparent for AS and CR; the AS was 41% lower in the non-nesting *vs* the nesting season, whereas the CR was 66% greater (Table 2; Fig 2).

**Table 2:** Collective activity space (95% PVC) and core range (50% PVC) areas (pooled across individual satellite-tagged tiger sharks) within the Galapagos Marine Reserve.

		Number of sharks	Activity space (km^2^)	Core range (km^2^)
Season	Nesting	11	6,500	356
	Non-nesting	6	3,827	1,046
Size range	Large (>300 cm TL)	3	3,324	422
	Medium (200–300 cm TL)	10	7,088	406
	Small (<200 cm TL)	1	782	179

#### Habitat usage at study sites

**Fig 3:**
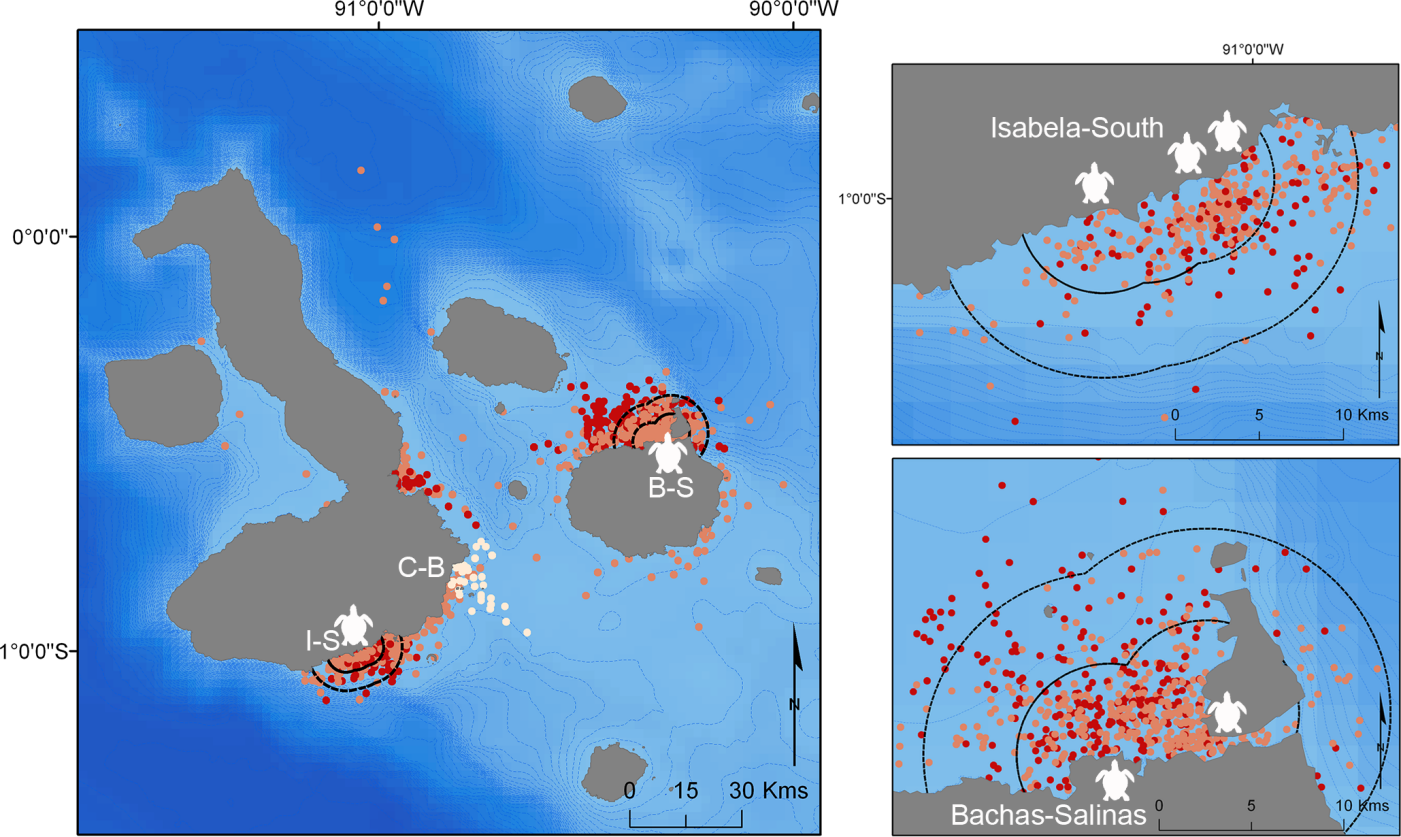
12-hourly estimated positions provided by SSM by shark size. Colours indicate three size classes of tiger sharks (large = red, medium = orange, small = white). Black dashed lines indicate the 5 and 10 km buffer areas around the study sites (I-S = Isabela-South, C-B = Cerro Ballena, B-S = Bachas-Salinas) and sea turtle nesting beaches (white sea turtle icon). Local bathymetry is displayed by 100 m isobaths (blue dashed lines). Right panels show zoomed areas of the study sites of I-S (upper) and B-S (lower).

Of the total time that medium and large sharks were tracked within the GMR, 80% of the time was spent within 10 km, and around half of the time within 5 km, of the sea turtle-nesting beaches (Fig 3). Almost all (99.33%) of the time spent within the GMR corresponded with resident behaviour. In contrast, the only small shark that provided a satellite track did not approach the nesting locations, and remained in the vicinity of the third study site, Cerro-Ballena, where it had been tagged (Figs 2C and 3). There were no significant differences, however, between medium and large sharks in the proportion of time spent within 5 or 10 km of nesting beaches ($\chi_{\left[2\right]}^{2}$ = 1.93, *p* = 0.38).

**Fig 4:**
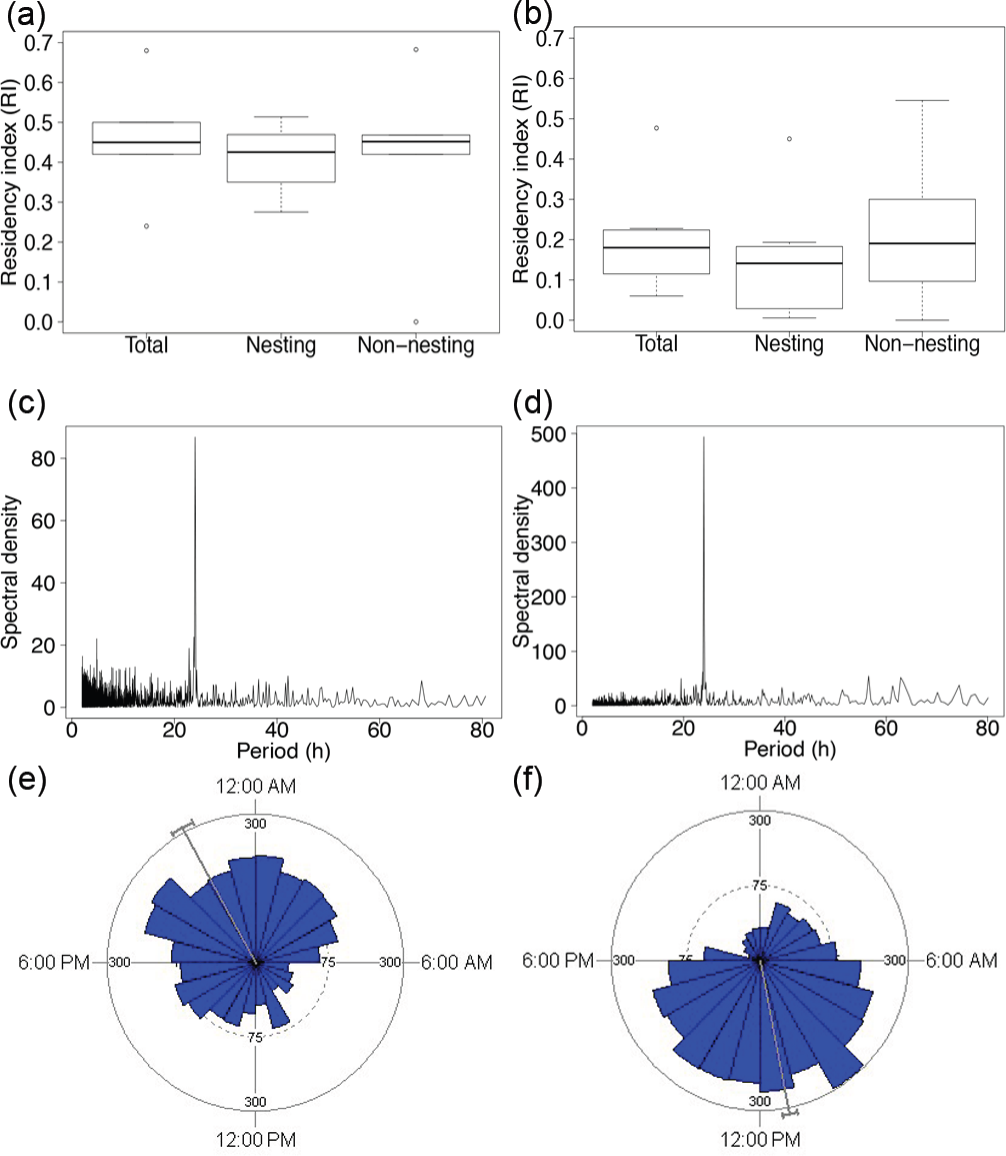
Patterns of residency and diel occurrence of acoustic-tagged tiger sharks. Left panels refer to Bachas-Salinas and right panels to Cerro-Ballena. The top panel (a, b) shows residency index (RI, the total number of days a shark was detected divided by the number of days that the shark was monitored by the receivers) for the total monitored time (Total) and per season (Non-nesting and Nesting); the middle panel (c, d) shows Fast Fourier Transformations (FFT) of the number of hourly detections, with peaks indicating periods of dominant cycles; and the lower panel (e, f) shows daily detections of tiger sharks; the circle represents a period of 24 hours and the length of each wedge indicates the number of detections within each hour.

None of the sharks tagged at Bachas-Salinas were ever detected by acoustic receivers at either of the other two locations, but two sharks (TS9 and TS13) tagged at Cerro-Ballena were detected briefly at Bachas-Salinas (S3 Fig). The residency index (RI) was not correlated with the TL of the sharks (*r*^2^ = 0.11021, *p* = 0.27). At both locations, the RI was very similar between seasons (Fig 4A and Fig 4B). Spectral analysis (FFT) revealed a strong diel cycle of use (Fig 4C and Fig 4D), with highly differentiated proportions of day *vs* night detections in the two locations ($\chi_{\left[1\right]}^{2}$ = 1685.2, *p* < 0.001). Detections of sharks at Bachas-Salinas occurred almost exclusively at night, while those in Cerro-Ballena were mostly restricted to daylight hours (Fig 4E and Fig 4F).

### Size structure and relative abundance at study sites

**Fig 5:**
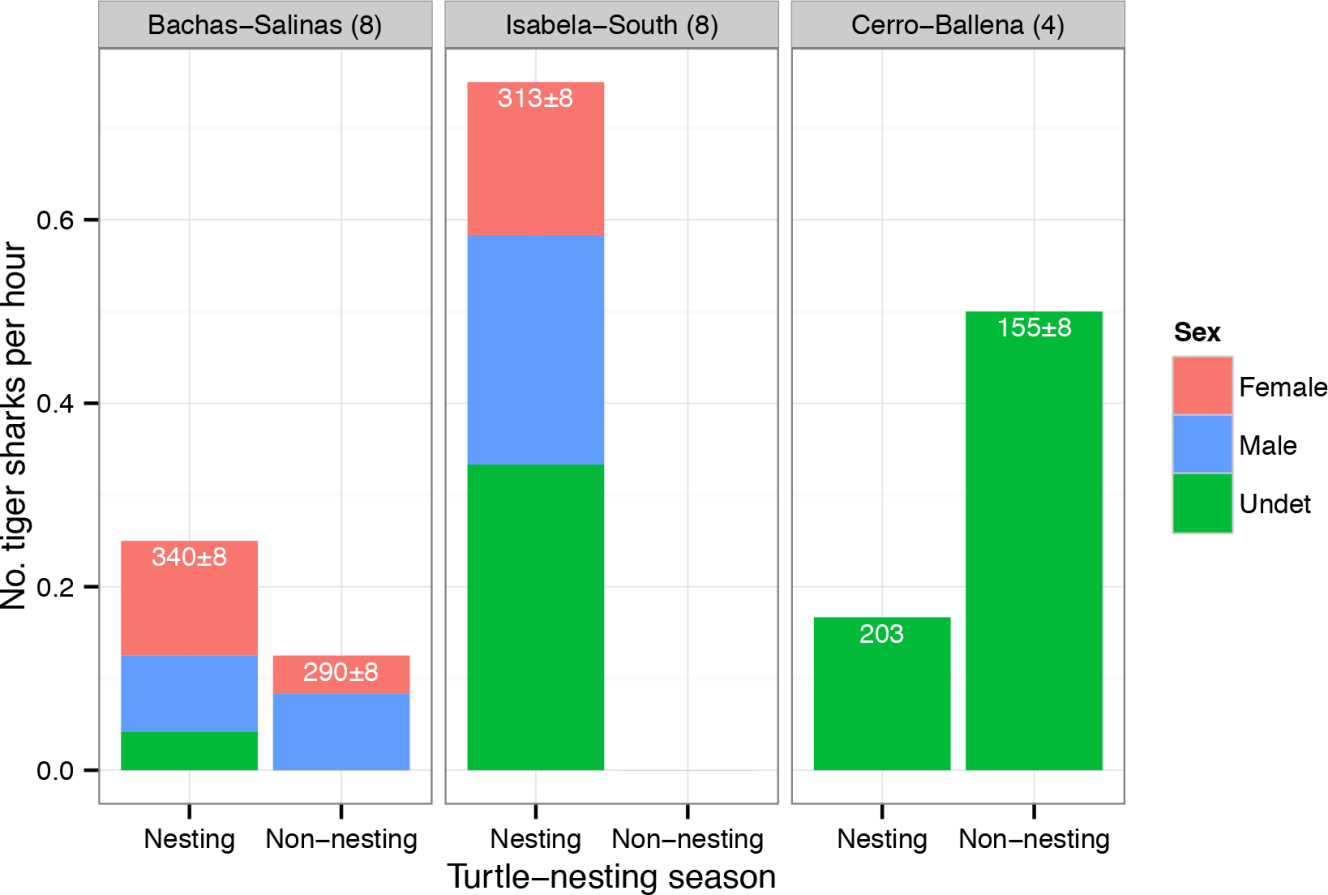
Relative abundance of tiger sharks at the three study sites. Number of individual tiger sharks per hour by sex recorded by the stereo-BRUVs in the nesting or the non-nesting season for turtles. The number of camera deployments at each site is reported in parentheses. The average TL ± SE (cm) of the sharks recorded at each site is given at the top of each bar.

Twenty tiger sharks (13 females, 7 males) were captured and tagged (Table 1) and another 22 sharks (8 female, 6 male, 8 undetermined) were recorded by stereo-BRUVs (Fig 5; S1 Table). The cMaxN counts of tiger sharks in the stereo-BRUVs depended on the season and the location. The seasonal pattern of counts differed significantly among locations (Fisher’s exact test, *p* = 0.01); specifically, more tiger sharks were recorded in the nesting season than in the non-nesting season at Bachas-Salinas and Isabela-South (none were detected at Isabela-South during the non-nesting season), whereas the reverse was true for Cerro-Ballena (Fig 5; S1 Table). There was no evidence for any differences in sex ratios among locations (Fisher’s exact test, *p* = 0.67; S1 Table).

The lengths of captured tiger sharks ranged from 140–383 cm TL and those observed by stereo-BRUVs ranged from 102–416 cm TL (S4 Fig). The mean TL of captured sharks was 247 cm, and that of sharks observed by stereo-BRUVs was 291 cm; although these means were not significantly different (*t*_40_ = 1.93, *p* = 0.06).

**Fig 6:**
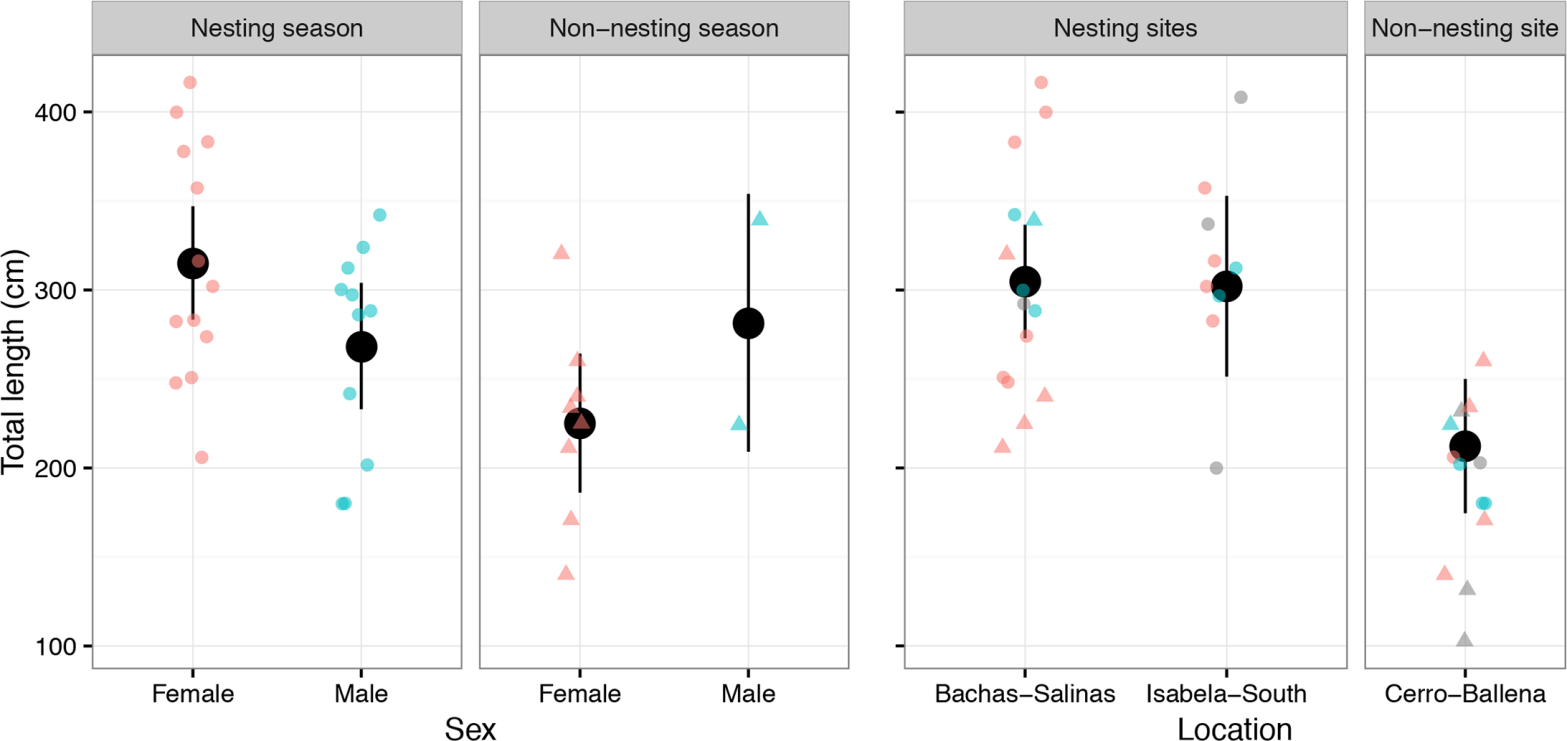
Total length (TL) of tiger sharks tagged and observed by stereo-BRUVs. Total length is shown as raw data values and as means (black circles) with 95% confidence intervals for males (blue) *vs* females (red) in either the nesting season (circles) or the non-nesting season (triangles), and at each of the three study locations.

There was weak evidence for an interactive effect of sex and season on the average lengths of sharks observed in the stereo-BRUVs (*F*_1, 28_ = 3.74, *p* = 0.06); females recorded were 53 cm longer on average in the nesting *vs* non-nesting season (*F*_1, 28_ = 4.56, *p* = 0.08), whereas the average lengths of males did not differ significantly between the two seasons (*F*_1, 28_ = 0.68, *p* = 0.42; Fig 6). A greater range of lengths was observed among the 21 females (140–416 cm TL) than the 13 males (180–342 cm TL), though there was no significant difference in variances between sexes (Levene’s test F_1, 28_ = 2.28, *p* = 0.37; Fig 6).

All but one shark either captured or recorded by stereo-BRUVs at Bachas-Salinas and Isabela-South were of medium or large size, whereas only small- and medium-sized sharks were observed at Cerro-Ballena (S71 Table). Accordingly, the mean lengths of sharks differed significantly among locations (*F*_2, 35_ = 9.43, *p* = 0.0007; Fig 6). There was no significant difference in mean lengths between the two nesting locations (ANOVA contrast of Bachas-Salinas *vs* Isabela-South, *F*_1, 28_ = 1.05, *p* = 0.31) but sharks observed at the non-nesting location of Cerro-Ballena were on average smaller (mean TL ± SE: 196.6 ± 15.4 cm) than those observed at the two nesting locations (308.25 ± 10.8 cm), and this contrast was significant (*F*_1, 28_ = 19.6, *p* < 0.001; estimated difference in means of 111.7 cm, 95% CI 70.6–152.7; Fig 6).

## Discussion

Tiger sharks tagged displayed strong philopatric behaviour, with intense use of specific areas associated with green turtle-nesting beaches that could provide year-round reliable food sources. Overall, tagged tiger sharks spent a remarkable 93% of their total tracked time within the protected waters of the GMR (Fig 1). The high availability of prey (i.e., the presence of a large population of nesting and resident sea turtles) and a potential provision of suitable habitats for all life stages, supported by the wide range of sizes recorded, might explain this high residency. Additionally, only two individuals exhibited long-distance round-trip movements. These two sharks showed strong site fidelity to the turtle-nesting sites at which they were tagged, both returning at the beginning of the subsequent turtle-nesting season. We consider that the remoteness of the GMR and the habitats that it provides, which include reliable and predictable food sources for adult tiger sharks, may structure the population into a smaller spatial extent than might be expected due to the potential mobility of this species [9]. Similarly, Heupel and Simpfendorfer [58] suggested that high levels of isolation, particularly in large and productive reefs, might result in an increase in the residency of sharks at the Great Barrier Reef (GBR). This high residency may enhance the effectiveness of the GMR to protect tiger sharks, suggesting that the inclusion of healthy ecological communities that ensure high prey availability can improve the efficacy of protected areas in the conservation of highly mobile top predators. Indeed, spatially restricted populations of reef sharks (e.g., due to small-scale ‘triangle migrations’, *sensu* Chapman et al. [9]) elsewhere have been successfully protected by properly enforced MPAs, even when nearby areas are heavily fished [13,59].

We found strong evidence that medium and large tiger sharks are using turtle-nesting sites as feeding grounds, as has been documented in other tropical locations [29–31]. Here, this inference is supported by two key results. Firstly, the movements of medium and large tiger sharks at the GMR were closely associated with the turtle-nesting sites, even outside of the turtle-nesting season (Fig 2E). Seminoff et al. [32] and Parra et al. [35] found that some of the nesting sea turtles at the GMR remained in the vicinity of their nesting areas once the nesting season had ended. We speculate that the reduced occurrence of large sharks at this time of year might enhance predation opportunities for remaining individuals on resident sea turtles. Extended residency by tiger sharks would allow them to avoid long migratory movements with high energetic costs. Secondly, we observed daily visits by sharks to the turtle-nesting sites almost exclusively at night, when turtles would be most available and vulnerable (Fig 4E), as green sea turtles are nocturnal nesters [60]. Similarly, great white sharks (*Carcharodon carcharias*) have been found to target cape fur seals (*Arctocephalus pusillus pusillus*) at their island entry and exit points during times of low light [61,62].

Our observations of high residency and fidelity of tiger sharks to areas of high prey availability, with some individuals conducting broad round-trip migrations, are consistent with results obtained in other studies done in areas having similar characteristics. In Raine Island (Australia), Fitzpatrick et al. [29] and Hammerschlag et al. [31] found year-round residency at an important sea turtle-nesting area for the majority of observed tiger sharks. A similar pattern was observed at the French Frigate Shoals (Hawaii Islands, USA), where some individual tiger sharks were residents while others just visited the atoll during the season with higher availability of bird prey [26].

The availability of breeding sites at the GMR is another potential reason for mature female tiger sharks to remain resident there. If we assume that tiger sharks grow *ca*. 100 cm year^-1^ (following Afonso et al. [63] and Meyer et al. [64]), then at least six of the 42 individuals recorded in our study were young-of-the-year, although a high degree of variation on growth rates has been reported for this species [64]. There are at least three other recent records of newborn (< 100 cm TL) tiger sharks at the GMR (Schuhbauer and Pazmiño pers. comm.), indicating that tiger sharks actively breed and pup at the GMR.

Tagged juvenile tiger sharks (< 200 cm TL) displayed spatial segregation from larger individuals, although two juveniles were detected for short periods of time at the sea turtle-nesting sites. While we only recorded one satellite track within this size range to support this, most of our records of juvenile individuals (from acoustic receivers, tagging activities and stereo-BRUVs) occurred in an area with no turtle-nesting beaches (Cerro-Ballena, Table 1; Fig 2 and Fig 5). The pattern of use at this site, with daily daytime visits, suggests that this area might be used to forage by juveniles on diurnal prey that differs from that of adults (given ontogenetic diet expansion in tiger sharks [27,28]). Juvenile tiger sharks may also be competitively excluded by larger conspecifics, and/or may actively avoid areas with larger tiger sharks to limit their exposure to potential cannibalism [23]. Juvenile tiger sharks might use Cerro-Ballena as a daytime refuge from which to conduct foraging excursions at nearby nocturnal feeding grounds (e.g., Cuatro Hermanos islets or the various adjacent seamounts). Patterns of spatial segregation of size classes have been reported in other large sharks in feeding areas elsewhere, such as white sharks at seal-colony hunting grounds [65,66]. Juveniles possibly remain resident in the GMR year-round, as limitations on broader movements for juvenile tiger sharks have been previously documented elsewhere [25,47].

Our study had relatively small sample sizes, particularly in the case of the number of stereo-BRUVs deployed and the number and duration of the satellite tracks obtained for small tiger sharks. The resulting number of recorded sharks (44 sharks seen on video or tagged), together with the recorded relatively short tracking durations (median = 66.5 days), necessarily limits the extent of our inferences and ecological interpretations of the patterns observed. Moreover, we focused our sampling efforts at discrete locations where tiger sharks had previously been reported to occur, thus the data collected is not representative of the entire tiger shark population of the GMR. Clearly, it is desirable that additional stereo-BRUVs surveys and tagging efforts be implemented throughout the GMR to more extensively document spatial patterns in population structure and relative abundances of tiger sharks. It is also worth noting that tagging location may have an impact on habitat use results (i.e., high residency to sea-turtle beaches may be an artefact of tagging sharks near these areas, and not related to food availability for the sharks). However, our results suggest that tagging location was not the driver of habitat use patterns. In fact, of the six tiger sharks tagged at non-nesting sites, the majority (4/6) were not detected again at the tagging site, but was instead subsequently detected at the turtle nesting beaches. Indeed, all of our results indicate that the GMR is a high-use area for tiger sharks across all life-stages and for both sexes.

It is remarkable that the local abundances of such a large predator at this highly visited World Heritage Site have gone unnoticed until recently [18]. This may reflect a recent recovery of the tiger shark population in the GMR, perhaps following the arrival of migrant individuals that then remained because of the suitable environmental conditions, year-round predictable and abundant food sources, and low levels of fishing. A similar case, albeit at a much smaller scale than the GMR, has been described at Cocos Island, Costa Rica, where tiger sharks apparently arrived in 2007 and since became year-round residents [67]. The long-term residency of tiger sharks in specific areas may exert strong structuring effects on local communities and ecosystem dynamics [68,69], so our findings may provide relevant insights for the understanding of the ecosystem functioning of the GMR.

This is the first published study on patterns of movement and habitat usage of tiger sharks in the Galapagos Islands and Eastern Pacific. The isolation and unique nature of the GMR indicate that the patterns observed may differ to those exhibited by tiger sharks elsewhere. We acknowledge that further studies are needed, especially to identify the evolving status of the population at the GMR, to establish its size-sex structure and spatial relative abundances, and to evaluate the importance of this area as a nursery ground, along with any inter-annual variations. Collectively, our findings suggest that the establishment of properly enforced MPAs that protect suitable habitats and predictable food resources for both juvenile and adult marine apex predators, even at relatively small spatial scales, might play a key role in the conservation of their populations.

## Supporting information

S1 Fig **Map showing the study sites of (a) Bachas-Salinas, and (b) Isabela-South and Cerro-Ballena.** White sea turtle icons indicate the most important nesting beaches for green sea turtles in the area, according to Zárate and Dutton [40] and Zárate et al. [34]. Black crosses show the locations of SBRUV deployments, and black rectangles show the locations of acoustic receivers.
(PDF)

S2 Fig Frequency distribution of the time interval (in days) between subsequent detections of satellite locations obtained for tagged sharks. (PDF)

S3 Fig Chronology of acoustic detections for each of the acoustic-tagged sharks (TS1-TS20) by site (colour coded). (PDF)

S1 Table Total number of sharks recorded in the study. Sharks observed in each season, of each size class, and of each sex, at each location (the numbers observed by stereo-BRUVs and by capture are given in parentheses, respectively). Sampling effort was not quantified for captures. For stereo-BRUVs, effort varied among locations but was equal between seasons within each location; the number of stereo-BRUV deployments in each season is given in parentheses for each location.
(PDF)

S1 File Stereo-BRUVs data for the study locations. (CSV)

S2 File Acoustic detections of the sharks tagged with acoustic devices in the four VR2W receivers deployed at the study locations. (CSV)

S3 File Satellite positions of the sharks tagged with satellite devices. (CSV)
