# Supplementary material for: Residency and movement patterns of an apex predatory shark (Galeocerdo cuvier) at the Galapagos Marine Reserve
Source: PLoS One. 2017 Aug 22;12(8):e0183669. doi: 10.1371/journal.pone.0183669 (PMC5567640; doi:10.1371/journal.pone.0183669)
Supplement: S1 Fig — Map showing the study sites of (a) Bachas-Salinas, and (b) Isabela-South and Cerro-Ballena. White sea turtle icons indicate the most important nesting beaches for green sea turtles in the area, according to Zárate and Dutton [40] and Zárate et al. [34]. Black crosses show the locations of SBRUV deployments, and black rectangles show the locations of acoustic receivers. (PDF) [file pone.0183669.s001.pdf]

(a)

(b)

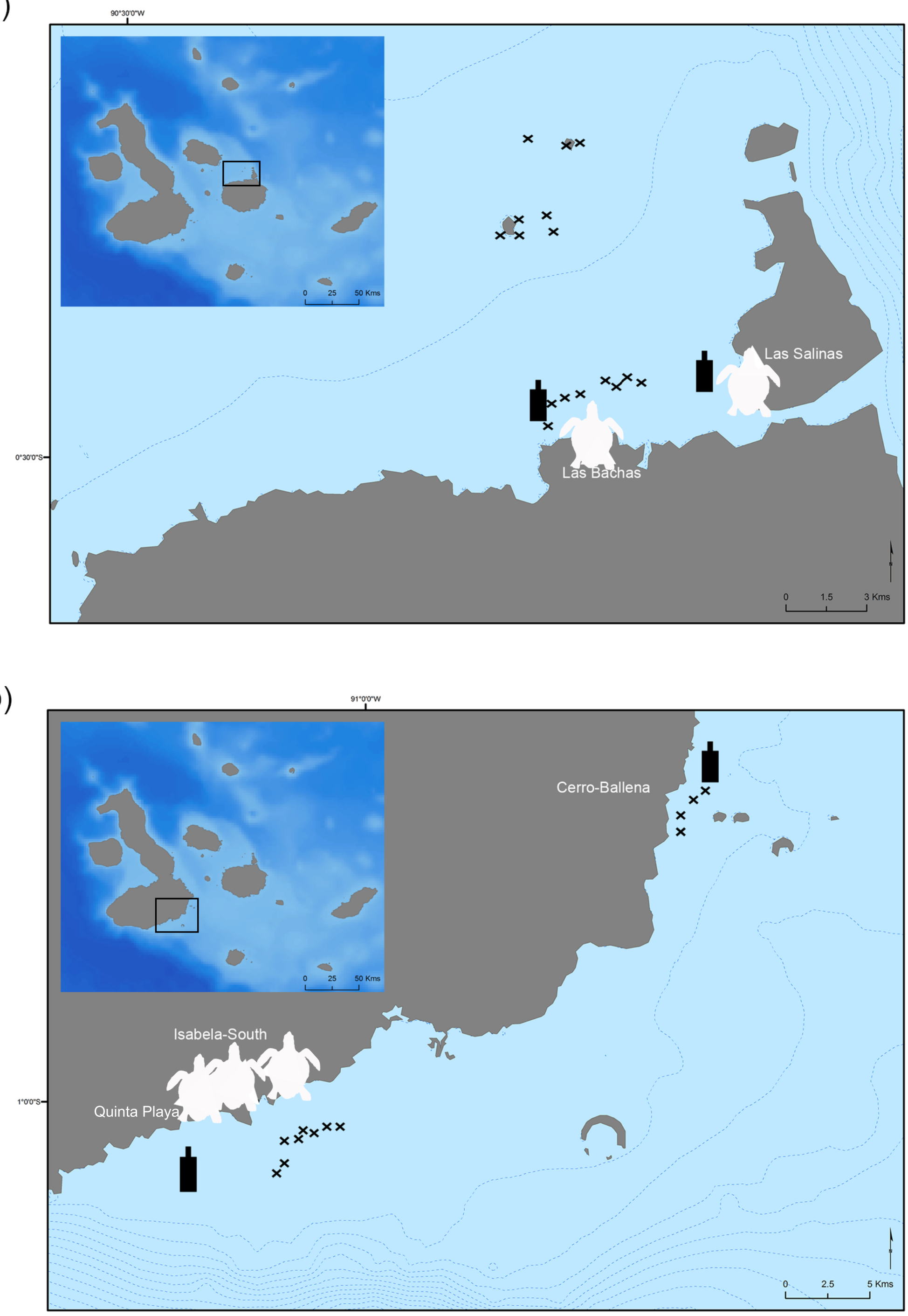

S1 Fig. Map showing the study sites of (a) Bachas-Salinas, and (b) Isabela-South and Cerro-Ballena.

White sea turtle icons indicate the most important nesting beaches for green sea turtles in the area, according to Zárate and Dutton (2002) and Zárate et al. (2013). Black crosses show the locations of SBRUV deployments, and black rectangles show the locations of acoustic receivers.
