# Supplementary material for: Residency and movement patterns of an apex predatory shark (Galeocerdo cuvier) at the Galapagos Marine Reserve
Source: PLoS One. 2017 Aug 22;12(8):e0183669. doi: 10.1371/journal.pone.0183669 (PMC5567640; doi:10.1371/journal.pone.0183669)
Supplement: S2 Fig — (PDF) [file pone.0183669.s002.pdf]

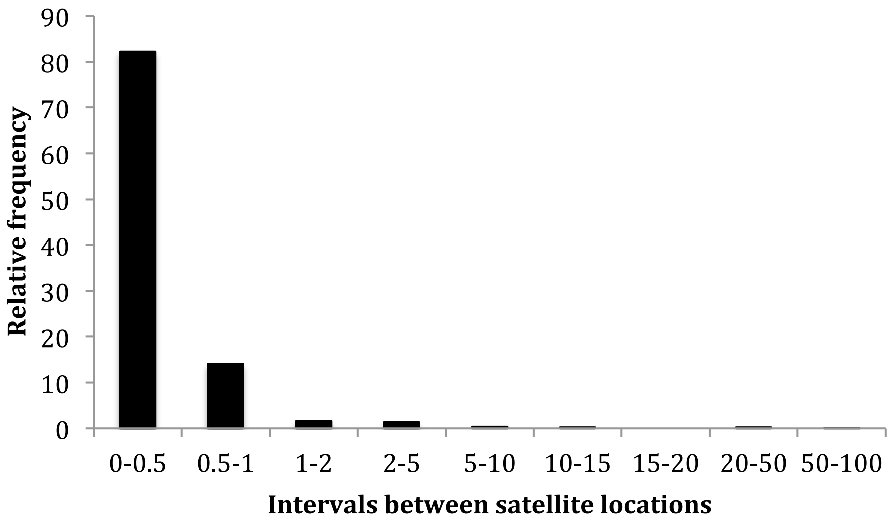

S2 Fig. Frequency distribution of the time interval (in days) between subsequent detections of satellite locations obtained for tagged sharks.
