# Supplementary material for: Residency and movement patterns of an apex predatory shark (Galeocerdo cuvier) at the Galapagos Marine Reserve
Source: PLoS One. 2017 Aug 22;12(8):e0183669. doi: 10.1371/journal.pone.0183669 (PMC5567640; doi:10.1371/journal.pone.0183669)
Supplement: S3 Fig — (PDF) [file pone.0183669.s003.pdf]

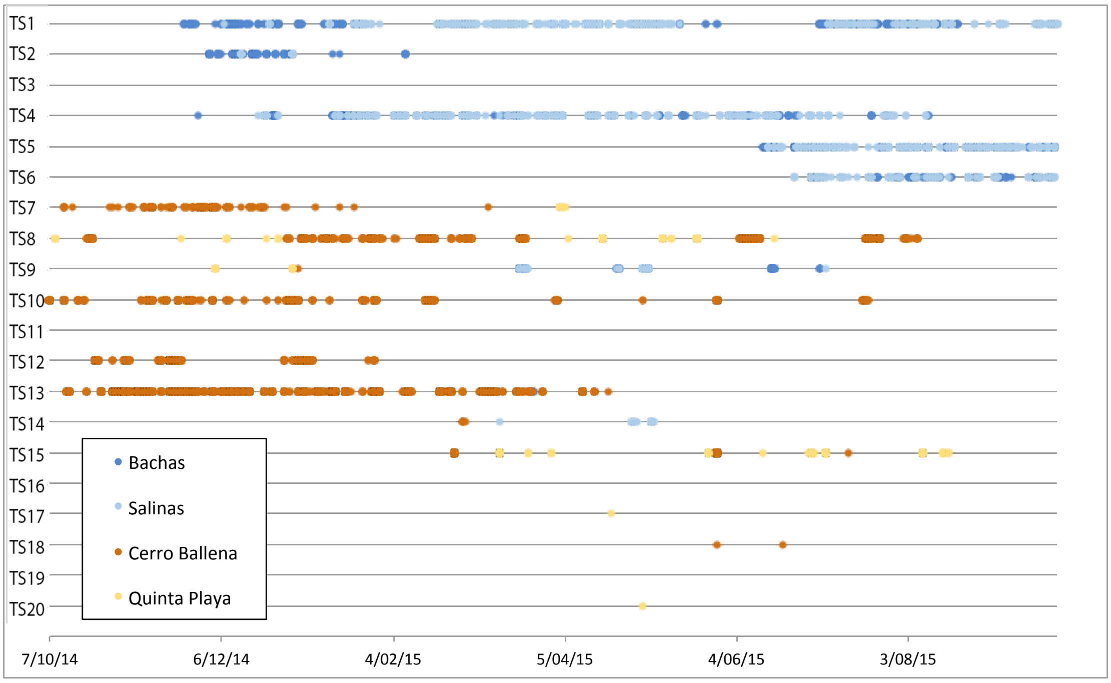

S3 Fig. Chronology of acoustic detections for each of the acoustic-tagged sharks (TS1-TS20) by site (colour coded).
