# Supplementary material for: Residency and movement patterns of an apex predatory shark (Galeocerdo cuvier) at the Galapagos Marine Reserve
Source: PLoS One. 2017 Aug 22;12(8):e0183669. doi: 10.1371/journal.pone.0183669 (PMC5567640; doi:10.1371/journal.pone.0183669)
Supplement: S1 Table — Sharks observed in each season, of each size class, and of each sex, at each location (the numbers observed by stereo-BRUVs and by capture are given in parentheses, respectively). Sampling effort was not quantified for captures. For stereo-BRUVs, effort varied among locations but was equal between seasons within each location; the number of stereo-BRUV deployments in each season is given in parentheses for each location. (PDF) [file pone.0183669.s004.pdf]

S1 Table. Total number of sharks recorded in the study.

Sharks observed in each season, of each size class, and of each sex, at each location (the numbers observed by SBRUVs and by capture are given in parentheses, respectively). Sampling effort was not quantified for captures. For SBRUVs, effort varied among locations but was equal between seasons within each location; the number of SBRUV deployments in each season is given in parentheses for each location.

	Location		
	Bachas-Salinas (16)	Isabela-South (8)	Cerro-Ballena (4)
Season: nesting	10 (6, 4)	14 (9, 5)	5 (1, 4)
Season: non-nesting	5 (3, 2)	0	8 (3, 5)
Sex: female	10 (4, 6)	6 (4, 2)	5 (0, 5)
Sex: male	4 (4, 0)	5 (2, 3)	4 (0, 4)
Size: large	7 (6, 1)	8 (6, 2)	0
Size: medium	8 (3, 5)	5 (2, 3)	7 (2, 5)
Size: small	0	1 (1, 0)	6 (2, 4)
TOTAL	15 (9, 6)	14 (9, 5)	13 (4, 9)
